# Investigation of the Possibilities of Wool Fiber Surface Modification with Copper Selenide

**DOI:** 10.3390/ma14071648

**Published:** 2021-03-27

**Authors:** Olga Belukhina, Daiva Milasiene, Remigijus Ivanauskas

**Affiliations:** 1 Faculty of Mechanical Engineering and Design, Department of Production Engineering, Kaunas University of Technology, 44249 Kaunas, Lithuania; olga.belukhina@gmail.com (O.B.); daiva.milasiene@ktu.lt (D.M.); 2 Faculty of Chemical Technology, Department of Physical and Inorganic Chemistry, Kaunas University of Technology, 44249 Kaunas, Lithuania

**Keywords:** wool fibers, copper selenide, surface modification, electrical resistance

## Abstract

A study of altering the conductive properties of wool fibers by applying copper selenide is presented. The researched modification of wool fibers was based on a two-stage adsorption-diffusion process. X-ray diffraction, scanning electron microscope, energy-dispersive X-ray spectrum, and Fourier transform infrared spectroscopy were performed to evaluate the morphological and physical characteristics of all Cu*_x_*Se-coated wool fibers. X-ray diffraction (XRD) data showed a single, Cu_0.87_Se (klockmannite), crystalline phase present, while Atomic Absorption Spectroscopy (AAS) and Energy Dispersive X-ray (EDX) analyses showed that the concentrations of Cu and Se in copper selenide coatings depend on the number of wool fiber treatment cycles. It was determined that a dense layer of Cu*_x_*Se grows through a nucleation mechanism followed by particle growth to fill out the complete surface. It was found that the conductivity of the coated wool fibers depends on the quality and density of the copper selenide coating, thus the resistance of electrically impermeable wool fibers can be reduced to 100 Ω by increasing the number of treatment cycles.

## 1. Introduction

The intensive development of modern technologies opens up new opportunities for material developers but at the same time places ever-increasing requirements on their properties of new materials. Many modern composite materials have more than one specific feature, such as antibacterial properties, resistance to fire, electromagnetic field shielding, etc.

Electromagnetic field (EMF) is a classical field produced by accelerating electric charges. Interference can sometimes occur from a natural source, like electrical storms, but more often than not it is usually a result of the actions of another device in close proximity.

Household appliances and other electrical devices, as well as power transmission networks and mobile antennas create electromagnetic fields, significantly increase the electromagnetic radiation in the human environment [1,2]. There have been many discussions about microwave ovens, computers, transmitters of various communication devices, and other EMF sources that influence people, especially children’s, health. Many studies on EMF influence on biological objects have been published. Now there is no doubt that EMF influence of a higher or lower degree exists on both microscopic and full organism levels.

Functional materials that can reduce or eliminate the negative environmental influence on human health are gaining importance. Therefore, solutions for modifying various materials so that they could contribute to the reduction or complete elimination of negative EMF effect on human health are being intensively sought. One group of such materials is functional textile materials modified with electrically conductive coatings. Conventional textile fabrics generally offer high electrical resistivity (>1010 Ω) [3,4,5]. Some researchers have already found a way for treating textile materials with organic conducting polymers, such as polyaniline, polythiophene, polypyrrole (PPy), etc. [6,7].

New special composite materials, with varying combinations of physical and chemical properties, can be obtained by using textile fibers of various nature modified with thin semiconductive or electrically conductive coatings of binary inorganic compounds. Recently, composite materials with metal chalcogenide layers have attracted interest for their potential applications in established areas, such as solar radiation control [8], photovoltaic devices [9], sensitive elements for gas sensors [10], photo electronic devices [11], as promising candidates for photocatalytic and electro catalytic applications [12], etc.

One of the options is a chemical surface modification of textile materials with thin coatings of a binary chalcogenide, such as copper selenide. Copper chalcogenides are denoted by a wide scope of application in various devices, such as solar cells, superionic conductors, photodetectors, photo-thermal converters, electroconductive electrodes, microwave shielding, coating, thermoelectric cooling, and optical filters. They also act as an optical recording material [13,14,15,16,17].

In our previous study, we found that EMF shielding effect was achieved by coating some textile materials with a layer of silver selenides [18]. Copper selenide was chosen as a cheaper option to silver because the electrical conductivity of copper is known to nearly match silver conductivity [19]. These types of materials are semiconductors with p-type conductivity. Several methods of precipitating copper selenide are mentioned in the literature (e.g., selenization [20], vacuum evaporation [21], solid state reaction [22], and chemical bath deposition [3,23]).

Developers of new composite materials face a challenging task of finding the way to use industrial waste as secondary raw material. The European Commission is increasingly highlighting the need to apply the principles of circular economy and resource efficiency to reduce the use of natural resources in the future [24].

Using industrial textile waste for the creation of new functional composites is a viable area for the development of sustainable technologies. The use of waste from the wool industry has particularly great prospects due to global wool production, which is estimated at 3.1 million tons per year [25]. Therefore, the incorporation of wool fiber waste into new functional composites is a highly topical and very promising challenge.

It is known that electrically conductive materials can also shield electromagnetic waves [26,27,28]. Therefore, forming electrically conductive layers on the surface of the wool fibers is a feasible solution. Modified industrial wool waste could be used for producing new composite materials with EMF shielding properties.

Nowadays, there are a lot of electrically conducting textiles that are produced by means of the insertion of metallic wires inside the yarns [29,30], by coating fibers with metals or by incorporating conductive fillers (e.g., carbon particles), but the processes of the production are not always appropriate for natural fibers and often are more expensive.

Wool is similar to polyamide in composition as they both have amide groups and chains. Previous work has shown [31] that electrically conductive copper selenide layers were formed on this polymer by adsorption/diffusion methods using potassium selenotrithionate salt as a selenium precursor. The main aim of this study is to investigate the possibilities of altering the conductive properties of wool fibers by applying copper selenide.

## 2. Materials and Methods

### 2.1. Materials

The 100% wool yarns (Nm 30/1 (or 33.3 tex)) made from mercerized 19.5 µm dyed wool fibers (white colour) (Suedwolle Group Italia S.p. A., Bijela, Italy) were investigated. All of the fiber samples were 1 m.

### 2.2. Treatment Methods

Before the treatment process, the investigated wool fiber samples were boiled in distilled water for 30 min to improve the adhesion of the copper selenide layer on the fibers’ surface. Then, the fibers were dried and kept in a desiccator with CaCl_2_ for 24 h. Experiments were repeated cyclically. At first, fiber samples were treated for 90 min with an aqueous solution of K_2_SeS_2_O_6_ (0.1 mol/L) at 60 °C and pH = 2.15. Salt of potassium selenotrithionate, K_2_SeS_2_O_6_, was prepared according to the method reported previously [32]. Pellets of Selenium (Se) (<5 mm particle size, ≥99.99% trace metal basis, acquired from Merck, (Merck Group, Darmstadt, Germany)), selenous acid, H_2_SeO_3_ (≥98.0%, Sigma-Aldrich, St. Louis, MO, USA), potassium pyrosulfite (K_2_S_2_O_5_) (≥96.0%, Chempur, Karlsruhe, Germany), and hydrochloric acid (0.1 mol/L, Fluka, Manchester, UK), were used for the synthesis of potassium selenotrithionate. Further, selenized wool fibers were treated for 10 min with Cu (I/II) salts solution at 80 °C so that to form a thin coating layer of Cu*_x_*Se crystals. The Cu (I/II) salts solution was made from crystalline CuSO_4_, hydroquinone and distilled water. Then, the samples were rinsed with distilled water and dried for 24 h. After each treatment cycle, the samples were conditioned and weighed to evaluate the mass change.

### 2.3. Investigative Methods

The mass of the investigated wool fiber samples was measured with AB104-S Analytical Balance (Mettler-Toledo (Switzerland) GmbH, Greifensee, Switzerland) featuring a measurement range of 110 g ± 0.1 mg, scale interval 0.1 mg, and error (0 ± 0.1) mg. The change in the mass of the sample *D_m_* is calculated by the formula:

(1) $$\Delta m=((m_{n}-m_{0})/m_{0})\times 100\%$$

where *m*_0_ is the mass of an untreated sample (mg); *m_n_* is the mass of the sample after treatment (mg); *n* is the number of the treatment cycle.

The total amount of copper and selenium in the wool samples was determined by using the AA–7000 Shimadzu (Tokyo, Japan) atomic absorption spectrometer (while using *λ* = 325.1 nm and *λ* = 196.0 nm) equipped with an electrode-less discharge lamp and air-acetylene flame. For the conditions described above, the sensitivity of the AAS method was ~0.02 µg/mL copper and ~0.5 µg/mL selenium for 1% absorption [33]. Before the analysis, the treated wool samples with coatings of copper selenide crystals were mineralized with concentrated HNO_3_.

Measurement of the electrical resistance of the investigated fibers was carried out by using a digital multitester PeakTech^®^ 3695 (PeakTech Prüf- und Messtechnik GmbH, Ahrensburg, Germany) (0.1 μA–10 A) and a DC Power Supply HY5003 (adjustable voltage 0–50 V), input voltage 50 Hz. The ampere meter we used was DPM type/model DT9205A (DPMSolid Limited Sp.k., Kowanowko Poland). The universal digital meter featured a power battery of 9 V and a fuse of F 500 mA/250 V. The treated fibers were clipped between two crocodile connectors connected to the DMM and stretched with a weight of 10 g. The connectors were 20 mm apart. Twenty measurements were performed for each sample.

The measurements were made at 5 A constant current, and the resistance was recalculated by using the formula:

(2) $$R=\frac{U}{I}$$

where *U* is the difference in potential along the object (calculated in volts); *I* is the current flowing through the object and calculated in amperes.

Tensile properties were determined using standard testing machine Zwick/Z005 (sensor KAP—Z 50N) (ZwickRoell GmbH & Co., Ulm, Germany) and standard methods (experimental length—500 mm, clamp movement speed—500 mm/min). All testing parameters were chosen according to standard EN ISO 2062:2009 [34]. Each stretch test in the group was repeated with 50 samples.

Cu*_x_*Se coatings obtained as described above were analyzed by XRD while using a D8 Advance diffractometer (Bruker AXS, Karlsruhe, Germany) with the software package DIFFRAC.SUITE (Diffract.EVA.V4.3., Bruker, Karlsruhe, Germany), a tube current of 40 mA, and source power of 40 kV. A silicon strip technology of a fast counting one-dimensional detector Bruker LynxEye, for recording diffraction patterns, and Bragg–Brentano geometry was used. Samples with Cu*_x_*Se coatings were scanned in steps from 5° to 70 ° in 2*θ* mode. The scanning speed was 6° min^−1^. The X-ray β-radiation of Cu-Kα was filtered with a 0.02 mm Ni filter. Computer programs of Search Match, Xfit, and ConvX software packages were used for processing X-ray diffraction patterns of PA sheets with copper selenide films.

The crystallite size along a specific crystal plane was calculated by using Scherrer’s formula [35,36] based on the XRD data:

(3) $$D=\;\frac{k\lambda }{\beta cos\theta }$$

where, *D* is average crystallite size, *λ*—X-ray wavelength (1.54178 Å), *k*—the shape factor (*k* = 0.9), *β* is peak full-width at half-maximum (FWHM), and *θ* = Bragg’s angle.

The morphology was examined using a Quanta 200 FEG (FEI Compant^TM^, Eindhoven, The Netherlands) scanning electron microscopy (SEM) equipped. A secondary electron signal was used for imaging. Energy Dispersion Spectroscopy (EDS) imaging was performed by using QUANTAX EDS system with an Bruker XFlash^®^ 4030 detector and ESPRIT software (Bruker AXS Microanalysis GmbH, Berlin, Germany).

Perkin-Elmer FTIR Spectrum GX (Thermo Fischer Scientific, Waltham, MA, USA) spectrometer was used to obtain infrared spectra. Used scan speed was 0.2 cm·s^−1^, resolution 1 cm^−1^, and scan number was 16 times. Area peaks in spectra ΔS A·cm^−1^ were calculated using “Spectrum 5.0.1” software (Perkin-Elmer). A total of 2 mg of cut fibers and optically pure KBr were used to make sample pallets for Fourier transform infrared spectroscopy (FT-IR) analysis. Short, cut to 1–2 mm length, wool fiber segments were used. Then, 10 × 0.5 mm pallets were used inside a special holder. A wave range between 4000 and 650 cm^−1^ was used for pallet spectra.

## 3. Results and Discussion

### 3.1. Physical and Mechanical Measurements

A two-step adsorption/diffusion method for the deposition of a copper selenide coating on the surface of wool microfibers was proposed. The optimal conditions for each step were fine-tuned in research studies [31,37]. In the first step, divalent selenium anions adsorb and diffuse into the subsurface of wool fibers. The first sign of a successful course of the treatment process is the visible change in wool fiber colour, from white to slightly reddish, after the seleniumization. The change in colour indicated that part of the diffused colorless anions of SeS_2_O_6_^2−^ slowly decompose and lead to the formation of secondary ions, such as SeSO_3_^2−^ and Se_2_S_2_O_6_^2−^ [38], in wool fibers.

**Figure 1:**
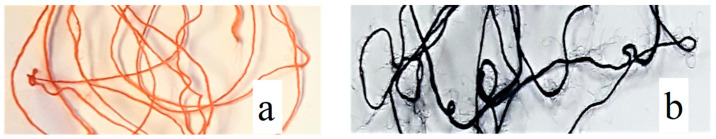
Examples of modified wool samples: (**a**) Slightly reddish after seleniumization, (**b**) black after complete formation of the copper selenide coating layer.

During the second step, the seleniumized wool fibers reacted with Cu(I/II) ion solution. The changed colour of the wool fibers, from slightly reddish to gray, indicates the formation of Cu_x_Se crystals on the surface of wool microfibers. By increasing the number of treatment cycles, the fiber samples become completely black (Figure 1).

**Figure 2:**
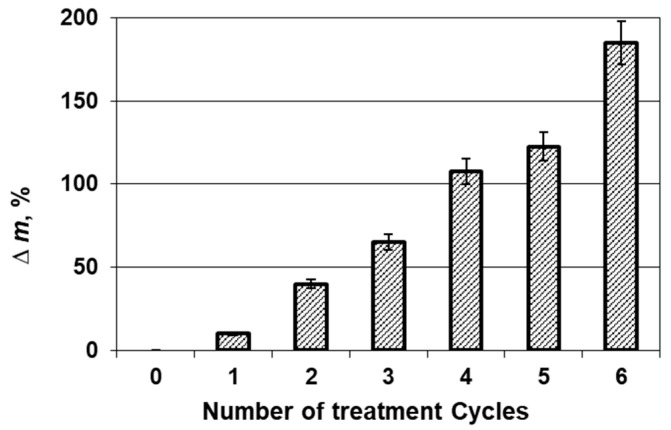
Influence of the treatment cycle number on the fibers mass change.

The formation of a crystal coating on the surface of the wool samples was also confirmed by an increase in the mass of the investigated wool fibers after each treatment cycle as well as electrical conductivity of the wool fibers. A significant increase in the sample mass was observed after each treatment cycle (Figure 2). The mass of the samples increased by 15 % already after the first treatment cycle and it continued increasing substantially after further cycles.

In order to improve the quality of the copper selenide layer, the duration of aggressive chemical treatment has to be increased and thus can cause damage to the fibers. For this reason, the influence of the modification process on the mechanical properties of treated wool fibers was investigated. The obtained standard tensile test results show that the modification reagents selected and the process conditions applied were very suitable for processing natural protein fibers. According to the results presented in Table 1, the values of breaking tenacity of the investigated wool did not diminish after the first two cycles of modification. A slight increase in the tensile strength of the treated wool fibers was observed. Although the change is non-significant change, it can be assumed that the growing coating crystals bind the micro hair of wool fibers. This is confirmed by the obtained elongation at the break value. After the third treatment cycle, the strength of the treated wool fibers started decreasing slightly, but the obtained results are within the limits of error and remain close to the initial strength even after six treatment cycles. Meanwhile, the deformability of the treated wool fibers remains significantly higher.

**Figure 3:**
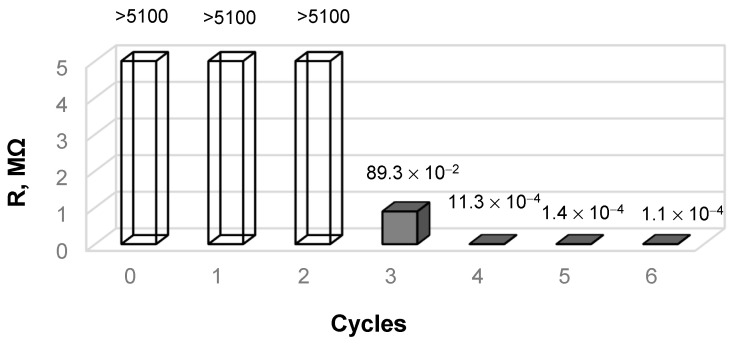
Resistance measurement.

According to the electrical resistance measurement results (Figure 3), the resistance of both untreated wool and the samples obtained after the initial treatment cycles is very high (>5100 MΩ), thus it could not be validly measured by the equipment used in this research. However, after the third cycle of the treatment process, the electrical resistance values of the tested samples reduced significantly to a measurable value (to an average of 89.3 kΩ).

The continuation of the treatment process and increased number of treatment cycles caused the electrical resistance of copper selenide-coated wool to decrease significantly: To an average of 1.13 kΩ after four cycles, to 140 Ω after five cycles, and to 100 Ω after six cycles.

### 3.2. Bulk Chemical Composition and XRD Characterization of CuxSe Coatings

X-ray diffraction, frequently abbreviated as XRD, is a useful technique used to evaluate minerals, polymers, corrosion products, and unknown materials. It is a non-destructive test method used to analyze the structure of crystalline materials. Phases are identified by comparison of the measured pattern with the entries in the reference XRD database. XRD analysis was conducted for the structural characterization of Cu*_x_*Se coatings obtained in the tests.

**Figure 4:**
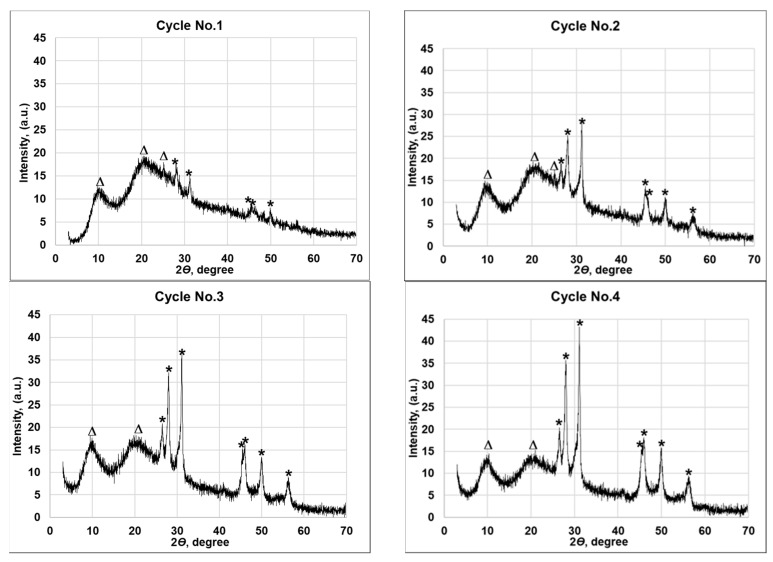

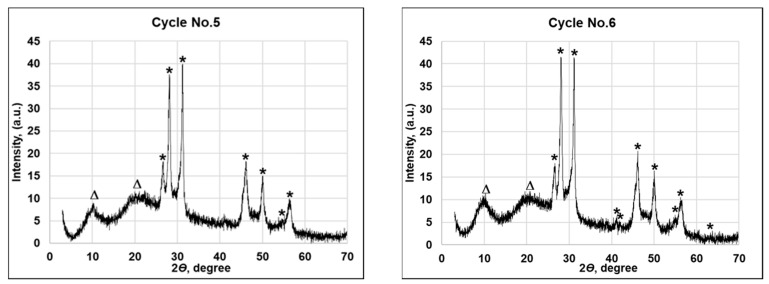
X-ray diffraction patterns of wool fibers with coatings of Cu*_x_*Se obtained with different numbers of cycles. Peaks were identified and assigned as follows: (*)—hexagonal klockmannite Cu_0.87_Se (JCPDS no. 83-1814); (∆)—wool fiber.

X-ray diffraction patterns were measured in the 5°–70° 2*θ* angular interval. Complex diffraction patterns were obtained due to polycrystalline nature of the Cu*_x_*Se layer along with the high degree of crystallinity of the wool fiber [39,40]. Joint Committee on Powder Diffraction Standards (JCPDS) reference templates and data available in the literature [41] were used for the analysis of XRD results. X-ray diffraction results are shown in Figure 4 and the corresponding peak values are listed in Table 2. Supplemental Information Table S1 shows the only single phase of Cu*_x_*Se—hexagonal klockmannite Cu_0.87_Se (JCPDS no. 83-1814) [42]. As illustrated in Figure 4, there are only two peaks attributable to the wool fibers visible at 10.24° and 20.93° in all diffraction patterns of copper selenide coatings. Meanwhile, the increasing number of deposition cycles increases the number of Cu_0.87_Se peaks and their intensity. The two diffraction peaks of the wool fibers are the most dominant after the first deposition cycle at 2*θ*—10.24°, 20.93°, and one lower intensity at 25.19°. They significantly exceed the copper selenide peaks. A higher number of deposition cycles increases the intensity of copper selenide peaks, which exceed the intensity of the wool fiber peaks. Meanwhile, the increasing intensity of the selenide layer overshadows the lowest intensity peak of the wool fiber at 25.19° already after the second cycle. Furthermore, the results presented in Table 2 show that the number of Cu_0.87_Se layer XRD peaks also increases, from seven after the first cycle to eleven after the last cycle. The increasing number of Cu_0.87_Se peaks and their intensity after each cycle indicate the increased concentration of this phase in the copper selenide layer. The same is confirmed by the bulk chemical analysis, which was performed on the selected wool fiber samples after each cycle using atomic absorption spectroscopy, with the results presented in Table 3. The detected amounts of copper and selenium are seen to have gradually increased from 0.356 to 5.754 mmol/g and from 0.56 to 8.84 mmol/g, respectively, with the increasing number of cycles.

Meanwhile, the molar ratio of Cu:Se in all coatings is similar to 0.64. However, this ratio is 0.87 in the copper selenide phase of hexagonal klockmannite Cu_0.87_Se. This ratio difference can be explained by the previous report [31], demonstrating that elemental selenium is formed as a consequence reaction of the decomposition product of SeS_2_O_6_^2−^ ion according to reactions (4) and (5):

Se_2_S_2_O_6_^2−^ + Cu^2+^ + 2H_2_O → CuSe + Se + 2H_2_SO_4_ (4)

SeSO_3_^2−^ + 2H^+^→ Se + H_2_O + SO_2_ (5)

The copper selenide formed and elemental amorphous selenium remain adsorbed within the wool fiber, whereas other ions dissolve back into the solution. However, amorphous selenium cannot be detected by X-ray analysis.

The peak analysis of XRD patterns was applied to determine the crystallite size. From the principal planes of the six most intense peaks 2*θ* at 26.58°, 28.09°, 31.15°, 46.13°, 49.98°, 56.23° of the phase Cu_0.87_Se 83-1814 of copper selenide coatings in Figure 4, crystallite sizes were estimated by Scherrer’s and tabulated in Table 2. It can be seen that the average crystallite size decreases after each cycle. It is likely that the formation of the smaller average crystal size is associated with the formation of a larger number of crystallization centers after each cycle. The increasing concentration of copper selenide crystals after each cycle results in multiple nucleation centers that yield a lower grain size, and, in turn, the lower concentration of copper selenide crystals results in fewer nucleation centers, thus yielding a higher grain size. This could be explained by the fixed amount of material used in crystallization during the reaction. The same amount of material divided on a larger number of nucleation centers would yield a smaller grain [43].

### 3.3. SEM and EDS Analysis of Cu_x_Se Coatings on Wool Fibers

**Figure 5:**
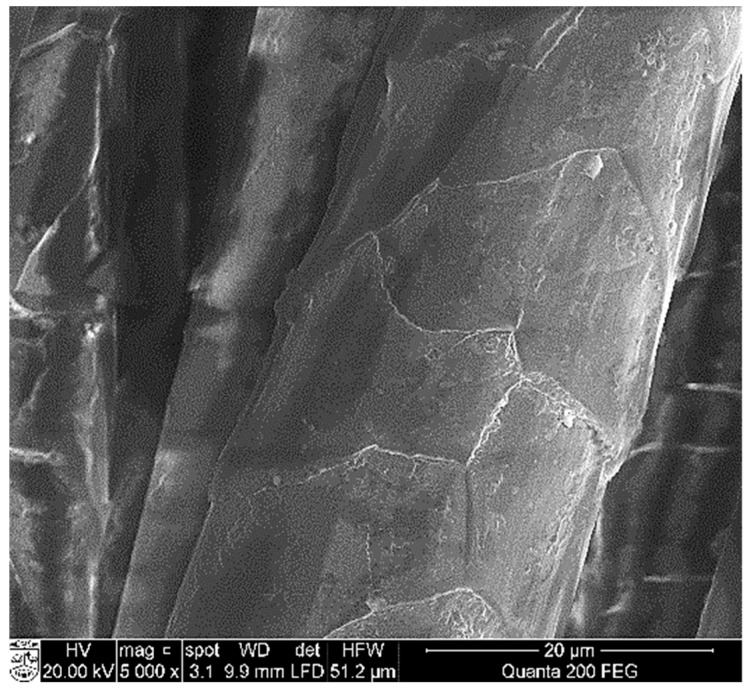
SEM image of wool fibers before treatment (magnification 5000×).

To estimate the formation of copper selenide coatings through adsorption/diffusion processes, the surface morphology and the elemental composition of the wool samples were examined by means of SEM and EDX analysis. Images of the wool fiber before treatment show a typical scaled structure of the wool surface (Figure 5) [44,45].

**Figure 6:**
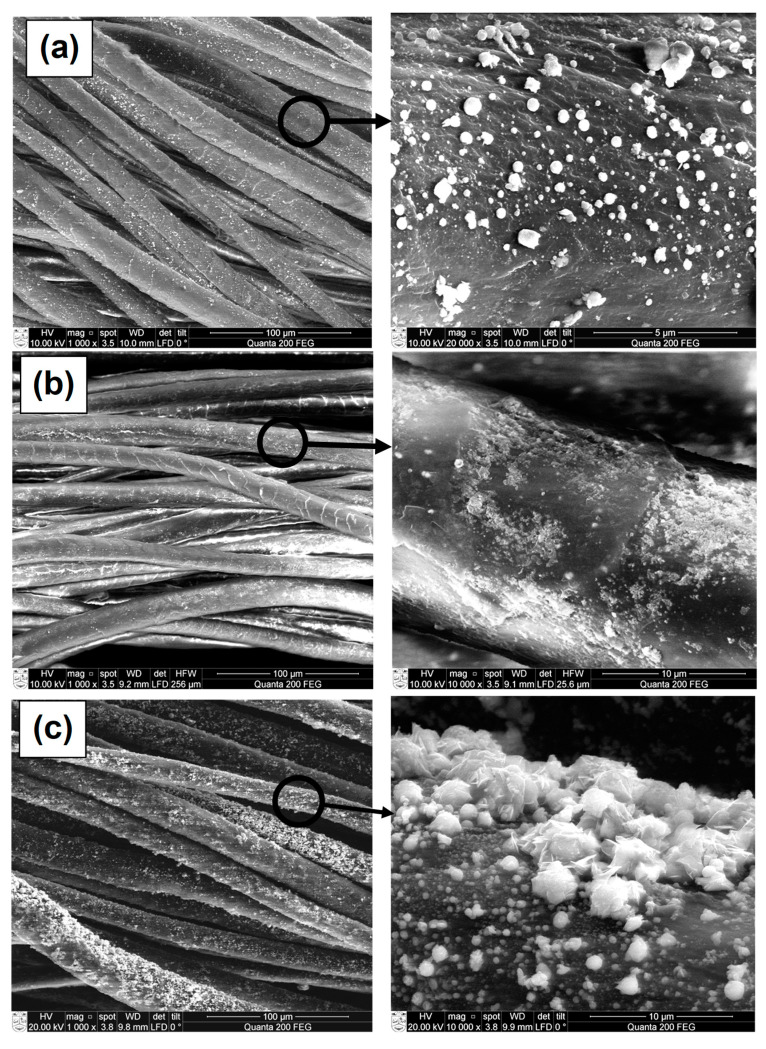

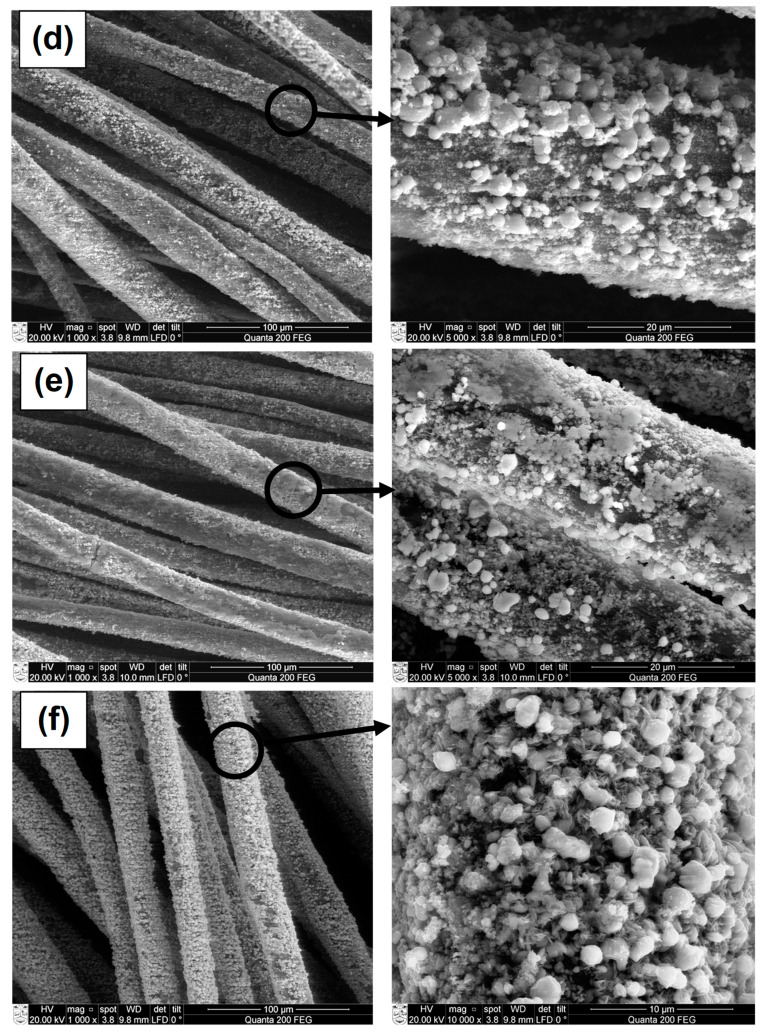
SEM images of copper selenide-coated wool fibers after different numbers of treatment cycles: (**a**) One (mag. 1000× and 20,000×), (**b**) two (mag. 1000× and 10,000×), (**c**) three (mag. 1000× and 10,000×), (**d**) four (mag. 1000× and 5000×), (**e**) five (mag. 1000× and 5000×), and (**f**) six (mag. 1000× and 10,000×).

Images of treated wool samples after a different number of treatment cycles are presented in Figure 6 (“mag.”—magnification). SEM analysis proved the formation of crystal coating on the wool fiber surface. It was observed that Cu*_x_*Se does not grow as a uniform film on the surface of treated fibers, but rather as a nucleation mechanism followed by particle growth to fill out the complete surface. With an increase in the number of treatment cycles, a clear change in the surface morphology of the wool fiber surface can be noticed.

The multiplication of crystal derivatives of various sizes is visible already after the first treatment cycle (Figure 6a). Most of the crystals formed are less than 1 µm in diameter. Our analysis showed that after the second and the third treatment cycles, the surface of the wool fibers was not yet fully covered (Figure 6b,c).

The prolongation of treatment duration by repeating the adsorption/diffusion process improved the density and the quality of the copper selenide layer. As shown in Figure 6d, the unbroken copper selenide layer of appropriate density forms on the surface of the investigated wool fiber only after the completion of the fourth cycle.

Not only mass change results but also the SEM images in Figure 6e,f show that the copper selenide crystal coating grows densely on the surface of wool fibers; however, the good quality of the coating is only achieved by the continuation of the treatment process to the required point.

**Figure 7:**
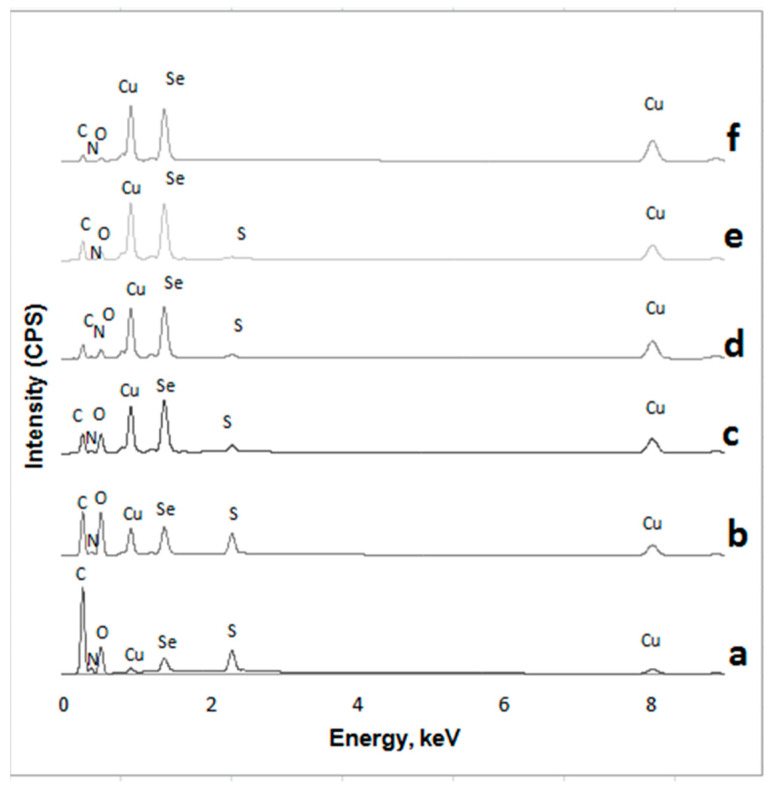
EDX spectra of treated wool samples after different numbers of cycles: a—1, b—2, c—3, d—4, e—5 and f—6.

The depth of sampling in EDX analysis is identified by the volume of interaction between the impact electron beam and the sample. It depends on the atomic number of the analyzed elements, as well as on the kinetic energy of electrons. The probed depth in EDX analysis reaches several micrometers. Figure 7 shows the EDX spectra gathered from the analyzed samples. The elemental concentrations obtained from the EDX analysis of the sample are listed in Table 4.

Several observations can be made on the grounds of the data obtained. First of all, the dominant peaks are associated with complex X-ray lines of Cu and Se, as well as strong C, O, and S signals in wool fibers. Other researchers [45,46,47] also reported the peaks corresponding to C, O, N, and S obtained in the analysis of pristine wool fibers. Sulfur is a characteristic component of wool protein keratin, which has a high content of the amino-acid cysteine (ca. 12% *w/w*) [45]. The peaks of Cu and Se originating from the Cu*_x_*Se coatings confirmed the formation of a well-defined thin coating layer.

Another important observation is that, after each subsequent treatment cycle, the C, O, and S peaks of the signal gradually decreased, at 0.276 keV, 0.54 keV, and 2.31 eV, respectively, whereas a marked increase was observed in the intensity of the Cu signals at 0.56 and 8.04 eV and the Se signal at 1.38 eV (Figure 7). The results presented in Table 4 show that the concentration of both copper and selenium increases monotonically; hereupon the concentration of other elements decreases. These findings distinctly suggest that the Cu*_x_*Se layer on wool fibers becomes thicker and denser after each subsequent cycle. These results confirm the impression based on the visual analysis of SEM images.

## 4. FTIR Analysis of Cu_x_Se-Coated Wool Fibers

**Figure 8:**
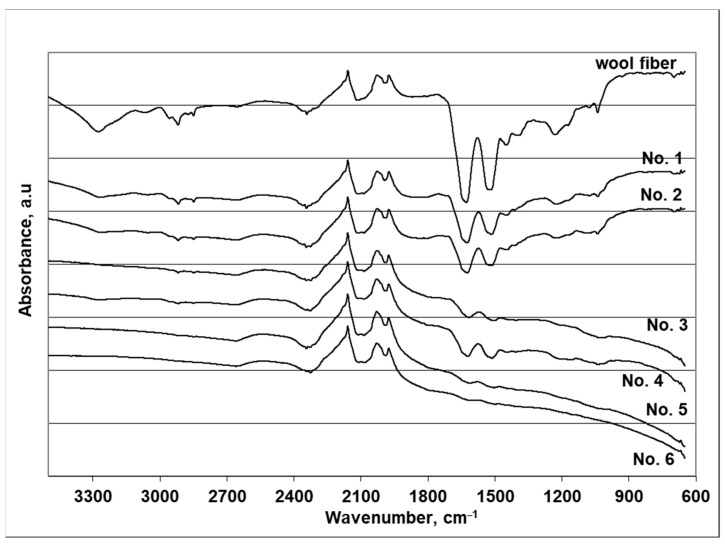
FTIR spectra of wool fiber and wool fibers with coatings of Cu*_x_*Se obtained with different numbers of cycles.

Fourier transform infrared spectroscopy is a very appropriate technique to estimate the changes in the wool structure. Therefore, infrared analysis of the discussed wool fiber samples was conducted to find out more details about the likely changes in the Cu*_x_*Se-coated wool fiber. The spectra of the pristine wool fiber and the six coated wool fiber samples are measured simultaneously. Simultaneous analysis provides excellent accuracy when comparing the light absorption intensities of the infrared rays of all wool fiber samples. FTIR spectra were measured using a compensation technique in the wavelength range 650–4000 cm^−1^. As seen from Table 5 and Figure 8, the spectra are similar. Although the IR spectra analysis did not spotlight any unusual peaks, after repeated deposition cycles the intensity of some peaks reduced significantly or they disappeared completely. The spectrum of uncovered wool fiber is chosen as a reading level. The FTIR absorption spectra of wool fibers show characteristic absorption bands attributed mainly to the peptide bond (–CONH–), which is fundamental to the structure of the polypeptide chain. As can be seen from Figure 7, the spectrum of uncoated wool fibers showed a broad absorption band at 3500–3200 cm^−1^, attributed to the overlapping of N–H and O–H [48]. Amide bands were also observed at 1629, 1517, and 1231 cm^−1^. The absorbance peak that appeared at 1517 cm^−1^ is assigned to the bending vibrations of N–H from amide II. The absorbance peaks at 1629 and 1231 cm^−1^ are assigned, respectively, to the stretching vibrations =C=O from amide I and of C–N from amide III. The peaks at 3276 and 3068 cm^−1^ in the absorption band at 3500–3200 cm^−1^ disappear after the second cycle, and the peak at 1231 cm^−1^ disappears after the third cycle. Meanwhile, two peaks of the highest intensity at 1629 and 1517 cm^−1^, attributed to the amines, remain after six cycles, but move slightly into the range of lower wavenumbers, and their intensity is reduced to a minimum (Table 5). The bands’ absorption peaks at 2961, 2919, 2874 and 2850 cm^−1^ in the range of 3000–2800 cm^−1^ might be associated with the symmetrical and asymmetrical stretching of the CH_2_ group [39,48]. After four cycles, all these peaks are no longer visible in the IR spectrum.

The peaks in the range of 1000–1200 cm^−1^ might be associated with vibrations of sulfur–oxygen groups of keratin [49]. There are three peaks in spectrums of wool fibers at 1172, 1140 and 1078 cm^−1^. Well-defined peaks at 1040 and 1172 cm^−1^ are assigned to the S=O stretching vibration originating from cysteic acid [40].

The trend of the change in peak intensity in the secondary amide and CH_2_ group is the same as in the previous peaks. Meanwhile, the peaks in the range 1900–2700 cm^−1^ cannot be attributed to the wool fiber, as natural wool fiber has no absorption peaks in this range [50]. As dyed wool fibers were used in the study, these peaks probably belong to the groups included in the dye composition [51]. In addition, as shown in Figure 8, the intensity and width of the peaks in this range do not change through-out the adsorption/diffusion cycles. According to the obtained FTIR analysis data, it can be stated that the wool fibers are degraded during the formation of the Cu*_x_*Se coatings. As a result, some of the main groups in the wool decompose and, consequently, the mechanical properties of the wool fiber change.

## 5. Conclusions

An adsorption/diffusion method was used for the formation of electrically conductive copper selenide coatings on wool fibers. In order to increase the electrical conductivity of the copper selenide coating, six cycles of adsorption/diffusion were used. The influence of the deposition conditions on the physical, structural, and morphological properties of wool fiber and Cu*_x_*Se coatings were investigated through AAS, SEM, EDX, XRD, and FTIR measurements after each cycle. The crystalline phases of wool fiber and copper selenide of Cu_0.87_Se (JCPDS no. 83-1814) were inferred from XRD data. AAS analysis of the Cu*_x_*Se coatings showed that the bulk molar Cu and Se concentrations increased with the increasing number of adsorption/diffusion cycles, whereas the Cu/Se molar ratio did not change. SEM results showed that the quality of the copper selenide crystal layer, formed on the surface of the investigated wool fibers, highly depended on the number of modification cycles. It was determined that a dense layer of Cu*_x_*Se did not grow as a uniform film on the surface of the treated fibers but, rather, through a nucleation mechanism, followed by particle growth to fill out the complete surface. The conductivity of the coated wool fibers changed with the number of cycles and the resistivity of wool fibers decreased from >5100 MW to 100 W. According to the obtained results, the unbroken coating of copper selenides with the appropriate density is formed on the surface of the investigated wool fiber only after the fourth cycle. After this cycle, the electrical conductivity of the wool fibers increased significantly. FTIR analysis showed that the internal structure of the wool fibers was partially damaged during the coating process; however, the tensile strength of the treated wool fibers did not decrease. These results show that the modification reagents and the process conditions selected were very suitable for processing natural protein fibers. The tested two-step adsorption/diffusion method for the formation of a copper selenide layer on the surface of protein fibers can be used for the modification of industrial wool waste, which can gain novel fields of application, thus lead to the creation of new insulating composites featuring EMF shielding properties.

## Figures and Tables

**Table 1:** Tensile characteristics of investigated wool fibers.

Characteristics		Treatment Cycle Number						
		0	1	2	3	4	5	6
Breaking tenacity, cN/tex	Min and max value, cN/tex	1.23/2.70	1.82/3.58	1.67/3.91	1.17/3.15	1.58/4.13	1.16/3.24	0.92/2.86
	X *, cN/tex	1.87	2.75	2.71	2.34	2.27	2.02	1.99
	Relative error, %	5.96	4.52	5.76	6.52	6.32	7.65	6.85
Elonga-tion at break, %	Min and max value, %	0.89/10.77	5.77/29.41	6.56/30.86	2.69/30.22	3.24/26.76	1.74/23.31	1.31/16.49
	X, %	5.47	17.23	17.80	14.31	12.94	9.67	7.38
	Relative error, %	14.04	9.37	10.58	13.09	12.94	17.02	13.33

* X—Averange (Arithmetic mean).

**Table 2:** XRD 2*θ* peaks and their ascription of a coating of Cu*_x_*Se formed on the wool fiber surface.

Number of the Treatment Cycle	Symbol in Figure 4; File Number of the Crystallographic Phase of Joint Committee on Powder Diffraction; Peak Positions 2*θ* (Degrees)	Average Size of Crystallite, nm
No. 1	(*) Cu_0.87_Se 83-1814 –28.09, 31.15, 45.41, 46.13, 49.98. (∆) wool fiber—10.24, 20.93, 25.19.	358.83
No. 2	(*) Cu_0.87_Se 83-1814—26.58, 28.09, 31.15, 45.41, 46.13, 49.98, 56.51. (∆) wool fiber—10.24, 20.93, 25.19.	327.31
No. 3	(*) Cu_0.87_Se 83-1814—26.58, 28.09, 31.15, 45.41, 46.13, 49.98, 56.51. (∆) wool fiber—10.24, 20.93,	323.73
No. 4	(*) Cu_0.87_Se 83-1814—26.58, 28.09, 31.15, 45.41, 46.13, 49.98, 56.51. (∆) wool fiber—10.24, 20.93.	318.39
No. 5	(*) Cu_0.87_Se 83-1814—26.58, 28.09, 31.15, 45.41, 46.13, 49.98, 56.23, 56.51. (∆) wool fiber—10.24, 20.93.	318.26
No. 6	(*) Cu_0.87_Se 83-1814—26.58, 28.09, 31.15, 41.13, 41.92, 45.41, 46.13, 49.98, 56.23, 56.51, 63.27. (∆) wool fiber—10.24, 20.93.	307.55

(*), (∆)—the marking of the identified peaks in Figure 4.

**Table 3:** Bulk elemental contents of copper and selenium in Cu*_x_*Se coating on wool fibers.

Treatment Cycles Number	Molar Concentration of Elements, mmol/g		Molar Ratio of Cu/Se
	Cu	Se	
1	0.356	0.560	0.64
2	1.288	2.011	0.64
3	2.117	3.316	0.64
4	3.292	5.179	0.64
5	4.516	7.105	0.64
6	5.751	8.840	0.65

**Table 4:** Surface elemental composition of the investigated treated wool fibers.

Number of Treatment Cycle	Elemental Composition in Weight Percent [Wt%]					
	C	O	N	S	Cu	Se
1	39.14	37.93	20.02	1.07	1.30	0.53
2	35.66	37.15	17.47	1.33	5.93	2.45
3	27.58	31.22	15.30	1.74	14.32	9.83
4	27.61	20.20	15.27	0.90	22.34	13.68
5	37.16	17.92	11.36	0.62	18.90	14.03
6	20.91	7.5	6.7	0.14	40.27	24.75

**Table 5:** The characteristics of functional groups bands in FTIR of wool fibers and wool fibers with coatings of Cu*_x_*Se.

Sample of Wool Fiber	Functional Group or Bond, to Which the Vibration Is Attributed and Band Position											
	N–H, O–H	Secondary Amide, Amide II Overtone	Methylene Symmetric C–H Stretching	Methylene Asymmetric C–H Stretching			Amide I Band (=C=O)	Secondary Amide N–H Bending, C–N Wagging	Amide Band (III)	(SO) Cysteic Acid	(SO) Cysteic Monoxide	
Uncoated wool fiber	3276	3068	2961	2919	2874	2850	1629	1517	1232	1172	1078	1040
No. 1	3273	-	2959	2918	2874	2850	1625	1515	1224	1172	1076	1040
No. 2	-	-	2961	2918	2874	2850	1625	1516	1226	1167	1080	1039
No. 3	-	-	2921	2921	-	2850	1616	1508	-	1165	-	1039
No. 4	-	-	-	2924	-	2848	1622	1513	-	1162	-	1043
No. 5	-	-	-	-	-		1615	1508	-		-	-
No. 6	-	-	-	-	-		1613	-	-		-	-

## Data Availability

The data presented in this study are available on request from the corresponding author.

## Supplementary Materials

The following are available online at https://www.mdpi.com/article/10.3390/ma14071648/s1, Table S1: Comparison between the JCPDS data (File No Cu0.87Se 83-1814) and experimentally observed values for copper selenide coatings formed via adsorption/diffusion method and structural parameters of these coatings.
