# Supplementary material for: Investigation of the Possibilities of Wool Fiber Surface Modification with Copper Selenide
Source: Materials (Basel). 2021 Mar 27;14(7):1648. doi: 10.3390/ma14071648 (PMC8037631; doi:10.3390/ma14071648)
Supplement: Supplementary file 1 [file materials-14-01648-s001.pdf]

materials

Supplementary Materials

Investigation of the Possibilities of Wool Fiber Surface Modification with Copper Selenide

Olga Belukhina [1], Daiva Milasiene [1] and Remigijus Ivanauskas [2,*]

[1] Faculty of Mechanical Engineering and Design, Department of Production Engineering, Kaunas University of Technology, Kaunas 44249, Lithuania; olga.belukhina@gmail.com (O.B.); daiva.milasiene@ktu.lt (D.M.)
[2] Faculty of Chemical technology, Department of Physical and Inorganic Chemistry, Kaunas University of Technology, Kaunas 44249, Lithuania
* Correspondence: remigijus.ivanauskas@ktu.lt; Tel.: +37-060-568-035

Citation: Belukhina, O.; Milasiene, D.; Ivanauskas, R. Investigation of the Possibilities of Wool Fibers Surface Modification with Copper Selenide. *Materials* **2021**, *14*, 1648. https://doi.org/10.3390/ma14071648

Academic Editor: Andrzej Katunin; Csaba Balázsi

Received: 19 February 2021
Accepted: 24 March 2021
Published: 29 March 2021

Publisher's Note: MDPI stays neutral with regard to jurisdictional claims in published maps and institutional affiliations.

Copyright: © 2021 by the authors. Licensee MDPI, Basel, Switzerland. This article is an open access article distributed under the terms and conditions of the Creative Commons Attribution (CC BY) license (http://creativecommons.org/licenses/by/4.0/).

Table S1. Comparison between the JCPDS data (File No $Cu_{0.87}Se$ 83-1814) and experimentally observed values for copper selenide coatings formed via adsorption/diffusion method and structural parameters of these coatings .

Number of the Treatment Cycle	2θ (°)	*d*-Spacing (Å)		Planes			Crystallite Size, *D* (nm)	Dislocation Density (δ) × 10^{12} Lines·m^{-2}	Strain, $\varepsilon \times 10^{-3}$ $Line^{-2}·m^{-4}$
		Observed Values	JCPDS Data	*h*	*k*	*l*			
No. 1	28.09	3.17	3.17	1	0	2	292.04	11.73	5.11
	31.15	2.87	2.87	0	0	6	327.74	9.31	4.10
	45.41	1.99	1.99	1	0	7	–	–	–
	46.13	1.97	1.97	1	1	0	453.59	4.86	2.03
	49.98	1.82	1.82	1	0	8	361.96	7.63	2.36
No. 2	26.58	3.35	3.35	1	0	1	331.87	9.08	4.75
	28.09	3.17	3.17	1	0	2	303.35	10.87	4.94
	31.15	2.87	2.87	0	0	6	353.16	8.02	3.82
	45.41	1.99	1.99	1	0	7	–	–	–
	46.13	1.97	1.97	1	1	0	318.72	9.84	2.90
	49.98	1.82	1.82	1	0	8	329.45	9.21	2.60
	56.51	1.62	1.62	1	1	6	–	–	–
No. 3	26.58	3.35	3.35	1	0	1	333.16	9.01	4.75
	28.09	3.17	3.17	1	0	2	314.49	10.11	4.76
	31.15	2.87	2.87	0	0	6	346.03	8.35	3.90
	45.41	1.99	1.99	1	0	7	–	–	–
	46.13	1.97	1.97	1	1	0	345.53	8.38	2.68
	49.98	1.82	1.82	1	0	8	324.7	9.48	2.64
	56.51	1.62	1.62	1	1	6	–	–	–
No. 4	26.58	3.35	3.35	1	0	1	328.04	9.29	4.81
	28.09	3.17	3.17	1	0	2	307.72	10.56	4.87
	31.15	2.87	2.87	0	0	6	341.92	8.55	3.95
	45.41	1.99	1.99	1	0	7	–	–	–
	46.13	1.97	1.97	1	1	0	344.22	8.44	2.69
	49.98	1.82	1.82	1	0	8	336.68	8.82	2.55
	56.51	1.62	1.62	1	1	6	–	–	–
No. 5	26.58	3.35	3.35	1	0	1	292.09	11.72	5.39
	28.09	3.17	3.17	1	0	2	311.17	10.33	4.79
	31.15	2.87	2.87	0	0	6	344.73	8.41	3.91
	45.41	1.99	1.99	1	0	7	–	–	–
	46.13	1.97	1.97	1	1	0	321.06	9.70	2.88
	49.98	1.82	1.82	1	0	8	315.89	10.02	2.71
	56.23	1.64	1.64	2	0	3	324.61	9.49	2.37
	56.51	1.62	1.62	1	1	6	–	–	–
No. 6	26.58	3.35	3.35	1	0	1	276.93	13.04	5.69
	28.09	3.17	3.17	1	0	2	320.46	9.74	4.66
	31.15	2.87	2.87	0	0	6	341.92	8.55	3.95
	41.13	2.19	2.19	1	0	6	–	–	–
	41.92	2.15	2.15	0	0	8	–	–	–
	45.41	1.99	1.99	1	0	7	–	–	–
	46.13	1.97	1.97	1	1	0	308.98	10.47	2.99
	49.98	1.82	1.82	1	0	8	293.56	11.60	2.92
	56.23	1.64	1.64	2	0	3	303.42	10.86	2.54
	56.51	1.62	1.62	1	1	6	–	–	–
	63.27	1.47	1.47	2	0	6	–	–	–
